# Impact of the COVID-19 Controlled Drugs and Substances Act exemption on pharmacist prescribing of opioids, benzodiazepines and stimulants in Ontario: A cross-sectional time-series analysis

**DOI:** 10.1177/17151635221126481

**Published:** 2022-10-12

**Authors:** Ann Chang, Shanzeh Chaudhry, Daniel McCormack, Tara Gomes, Anisa Shivji, Mina Tadrous

**Affiliations:** Leslie Dan Faculty of Pharmacy, University of Toronto; Leslie Dan Faculty of Pharmacy, University of Toronto; ICES, Toronto; Leslie Dan Faculty of Pharmacy, University of Toronto; Li Ka Shing Knowledge Institute, Unity Health, Toronto; Ontario College of Pharmacists, Toronto; Leslie Dan Faculty of Pharmacy, University of Toronto; Women’s College Hospital, Toronto, Ontario

## Abstract

**Background:**

Due to the coronavirus disease 2019 (COVID-19) pandemic, Health Canada issued an exemption to the Controlled Drugs and Substances Act (CDSA) on March 19, 2020, enabling pharmacists to act as prescribers of controlled substances to support continuity of care. Our study investigates utilization of the CDSA exemption by Ontario pharmacists with the intent to inform policy on pharmacist scope of practice and to improve future patient outcomes.

**Methods:**

We conducted a time-series analysis of pharmacist-prescribed opioid, benzodiazepine and stimulant claims data using Ontario Narcotics Monitoring System (NMS) data between January 2019 and December 2021. We used ARIMA modelling to measure the change to these classes of claims and to opioid claims containing quantities greater than a 30-day supply.

**Results:**

Postexemption, the average weekly number of pharmacist-prescribed opioid, benzodiazepine and stimulant claims rose by 146% (160 to 393 claims/week), 960% (49 to 515 claims/week) and 2150% (8 to 177 claims/week), respectively. There was a 2-week lag period between the time of announcement and the statistically significant increase in claims on April 5, 2020(*p* < 0.0001). The total number of claims for opioid quantities exceeding a 30-day supply decreased by 60%. Cumulative pharmacist-prescribed claims accounted for under 2% of the total NMS claims.

**Interpretation:**

Ontario pharmacists used the CDSA exemption but were prescribing at low rates. These findings suggest an effective change to pharmacy practice as the low rates show pharmacists used the exemption as a last line of defense. This may lead to further studies exploring treatment breaks during the COVID-19 pandemic and future changes to pharmacist scope to benefit patients.

## Introduction

The World Health Organization (WHO) declared the novel coronavirus disease 2019 (COVID-19) a pandemic on March 11, 2020.^1^ In response, the Canadian government enacted the COVID-19 Emergency Response Act and released a national health sector guidance document to facilitate delivery of care.^2,3^ In Ontario, a state of emergency was declared on March 17, 2020, resulting in widespread public facility closures.^4^ Unfortunately, the lockdown and resulting shift in health care delivery worsened existing gaps in the Canadian health care system, such as disruptions to primary care, drug shortages and rising numbers of opioid overdoses that proved difficult to mitigate.^5,6^ One major health care system concern was maintaining continuity of care in patients receiving any chronic treatment. Disruptions to primary care during the pandemic could put individuals at risk of withdrawal symptoms or serious adverse outcomes, particularly those chronically treated with controlled substances, as designated by the Controlled Drugs and Substances Act (CDSA), such as opioids, amphetamines and benzodiazepines.^7-9^ For example, patients requiring opioids to manage chronic pain may experience worsening pain, poor sleep patterns, functional decline and withdrawal as a result of cessation and may seek care at emergency departments, leading to inefficient utilization of health care resources.^10^ Additionally, patients receiving treatment for opioid agonist therapy (OAT) require regular interaction with the prescribing clinician and daily supervision in community pharmacies until they are cleared for take-home doses.^11^ This high-needs population is significantly at risk of disruptions in treatment and may experience significant clinical consequences.

**Knowledge Into Practice**

- In March 2020, during the coronavirus disease 2019 (COVID-19) pandemic, Health Canada implemented changes to the Controlled Drugs and Substances Act (CDSA), allowing pharmacists to verbally accept, adapt, extend and transfer prescriptions for continuity of care for patients using controlled substances (such as morphine, methylphenidate and lorazepam) who could not reach prescribers.
- In Ontario, facility closures and a shift to virtual care occurred, and emerging Canadian literature described the negative impact of the pandemic on patient access to health care. Community pharmacists took on additional responsibilities, such as higher workloads and additional sanitation protocols.
- Our findings suggest that pharmacists were judicious in their use of the exemption. This aligns with previous work assessing pharmacist prescribing and should be used to inform future decisions on health care policy and pharmacist scope of practice.

**Mise En Pratique Des Connaissances**

- En mars 2020, pendant la pandémie de la COVID-19, Santé Canada a apporté des modifications à la Loi réglementant certaines drogues et d’autres substances (LRCDAS), ce qui a permis aux pharmaciens d’accepter verbalement, d’adapter, de prolonger et de transférer des ordonnances pour assurer la continuité des soins aux patients utilisant des substances contrôlées (comme la morphine, le méthylphénidate et le lorazépam) et qui n’ont pas pu joindre leurs prescripteurs.
- En Ontario, des fermetures d’établissement et un passage aux soins virtuels ont eu lieu, et la littérature canadienne émergente a décrit les répercussions négatives de la pandémie sur l’accès aux soins de santé des patients. Les pharmaciens communautaires ont assumé des responsabilités supplémentaires, comme des charges de travail plus importantes et des protocoles sanitaires supplémentaires.
- Nos résultats suggèrent que les pharmaciens ont été judicieux dans l’utilisation de l’exemption. Cela correspond aux travaux antérieurs qui ont évalué la prescription par les pharmaciens et ces données devraient être utilisées pour éclairer les décisions futures concernant les politiques sur les soins de santé et le champ d’exercice des pharmaciens.

In response to this public health concern, Health Canada issued a temporary exemption to subsection 56(1) of the CDSA on March 19, 2020, allowing pharmacists to verbally accept, extend and transfer prescriptions for narcotics and targeted substances.^12^ These exemptions were initially intended to be temporary but were later extended to September 2026. The application of this federally enacted exemption varies at provincial and territorial levels due to different scopes of pharmacist practice.^13^ Some jurisdictions allow pharmacists to adapt controlled substances and opioids.^13^ Under this exemption, pharmacists are defined as those with a scope of practice that includes prescribing, such as adapting, extending or renewing drugs, including controlled substances.^12^ Transferring refers to a pharmacist sending a prescription to a different Canadian pharmacy for the purpose of having it filled at that pharmacy.^12^ Adapting a prescription refers to deprescribing, partially filling, or changing a patient’s dose or regimen for the prescribed drug.^14^ Provision of this exemption supported by complementary provincial regulatory changes enabled Ontario pharmacists to maintain access to controlled substances for patients in limited circumstances or when the prescriber is unreachable. Prior to the CDSA exemption, pharmacists were not authorized to adapt narcotic prescriptions.^14,15^ Prior studies conducted on this topic illustrate the need for pharmacists to prescribe controlled substances based on emergent patient needs or when there is difficulty in reaching prescribers. However, the rate of prescribing controlled substances by pharmacists in practice is currently unknown.^16,17^

A large emerging body of literature on COVID-19 describes the negative impact of the pandemic on hospital operations, medical education, health screening and patient access to health care.^18-22^ Importantly, the role of community pharmacists has shifted. Specifically, a qualitative study explored the role of Canadian pharmacists in providing care to patients with opioid use disorder (OUD) during the COVID-19 pandemic and found 3 major themes: optimization of opioid-related patient care, jurisdictional impact and awareness and education.^23^ Pharmacists were able to provide uninterrupted care to patients and participated in collaborative care with prescribers during the lockdowns, and the responsibility of pharmacists caring for those who receive chronic treatments was heightened.^23^ Although pharmacists played an integral role in providing continuity of care, many day-to-day operations, including workflow and workload, shifted.^23^ Pharmacists were faced with workflow challenges and inadequate staffing and time to spend with patients requiring additional interventions in care.^23^ However, current research on COVID-19–related changes to pharmacy scope and practice within the Canadian setting is limited. The objective of this study was to measure pharmacist utilization of the CDSA exemption by comparing pre- and postexemption opioid, stimulant and benzodiazepine prescriptions. This study captured renewing, adapting and extending prescriptions and did not capture verbal orders, delivery or transferring.

## Methods

### Study design

We conducted a cross-sectional time-series analysis using Ontario Narcotics Monitoring System (NMS) claims data. We included any opioid, benzodiazepine and stimulant claim and any opioid claim with a dispensed quantity exceeding a 30-day supply. Opioid and benzodiazepine classes were included due to ubiquity of use in the community setting and potential for withdrawal.^16^ Due to the concern of inappropriate prescriptions, opioid claims exceeding a 30-day supply were included as a patient safety parameter to provide insight on the appropriate use of this exemption, as long-term treatment with opioids is not recommended for nonchronic cancer pain due to tolerance and adverse events.^24^ Over-the-counter purchases of codeine were not captured within our analysis. We compared the changes to claims between the preexemption period, from January 6, 2019, to March 15, 2020, and the postexemption period, from March 22, 2020, to December 19, 2021.

### Data sources

The NMS is a prescription monitoring system that tracks the dispensing of controlled substances from Ontario retail pharmacies, regardless of payer.^25^ We obtained weekly NMS claims data that were reported on each Sunday of the month from ICES. NMS claims containing an Ontario College of Pharmacists (OCP) reference number in the prescriber ID reference field were designated as pharmacist prescribed, and all other claims were designated as total prescribed claims, which included pharmacists.^25^ NMS claims data were linked using unique encoded identifiers and analyzed at ICES.^26^ Use of this data is authorized under section 45 of Ontario’s Personal Health Information Privacy Act and does not require review by a research ethics board.

### Analysis

We reported the number of pharmacist-prescribed opioid, benzodiazepine and stimulant claims; the proportion of total prescribed opioid claims with dispensed quantities exceeding 30 days; and the total proportion of pharmacist-prescribed opioid, benzodiazepine and stimulant claims. We calculated 2 monthly averages for the number of claims between the preexemption period (January 6, 2019, to March 15, 2020) and the postexemption period (March 22, 2020, to December 19, 2021); the relative percent change for both the number and proportion of pharmacist-prescribed opioid, benzodiazepine and stimulant claims; and the average number of total prescribed claims. We also measured the time elapsed from the Sunday following the exemption to the week of the first increase in claims. We used Automatic Autoregressive Integrated Moving Average (ARIMA) modelling with step and pulse functions to determine the statistical significance of changes to the number and proportion of pharmacist-prescribed claims.^27^ Data analysis was conducted using Microsoft Excel and SAS software 9.2 for ARIMA modelling.

## Results

### Number of pharmacist-prescribed opioid, benzodiazepine and stimulant claims

**Figure 1:**
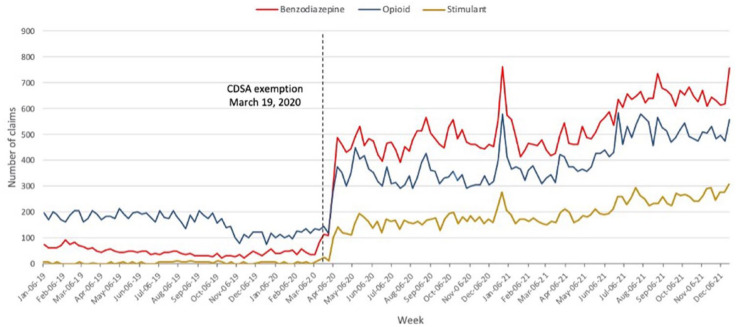
Weekly number of pharmacist-prescribed opioid, benzodiazepine and stimulant claims from January 2019 to December 2021

After the CDSA exemption was enacted on March 19, 2020, the number of pharmacist-prescribed opioid claims increased by 146%, from an average of 160 claims per week during the January 6, 2019, to March 15, 2020, period to 393 claims per week during the March 22, 2020, to December 19, 2021, period. The number of benzodiazepine claims increased by 960%, from an average of 49 claims per week during the January 6, 2019, to March 15, 2020, period to 515 claims per week during the March 22, 2020, to December 19, 2021, period. The number of stimulant claims increased by 2150%, from an average of 8 claims per week during the January 6, 2019, to March 15, 2020, period to 177 claims per week during the March 22, 2020, to December 19, 2021, period (Table 1, Figure 1). Interestingly, there was a 2-week lag period from March 22 (the first Sunday after the exemption was enacted) to April 5, 2020 (the shift in claims), prior to the increase in the number of opioid, benzodiazepine and stimulant claims. A statistically significant increase in the number of pharmacist-prescribed opioid, benzodiazepine and stimulant claims was found on April 5, 2020 (*p* < 0.0001) from the ARIMA step function (Appendix 1, available at www.cpjournal.ca). A peak in the claims data was observed on the week of December 27, 2020, with 582 opioid, 765 benzodiazepine and 269 stimulant pharmacist-prescribed claims dispensed (Figure 1).

**Table 1:** Weekly percent change to opioid, benzodiazepine and stimulant claims between pre- and postexemption periods

	Preexemption (January 6, 2019–March 15, 2020)	Postexemption (March 22, 2020–March 21, 2021)	% Change
Opioid claims (*n*)	160	393	146
Benzodiazepine claims (*n*)	49	515	960
Stimulant claims (*n*)	8	177	2150
Opioid claims >30 days (%)	5.7	2.3	–60

### Opioid claims with quantities exceeding a 30-day supply

**Figure 2:**
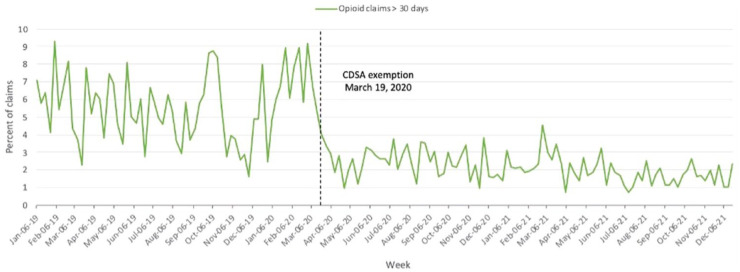
Weekly percent of prescriber-independent opioid claims exceeding a 30-day supply from January 2019 to December 2021

The proportion of total prescribed opioid claims with quantities exceeding a 30-day supply decreased by 60% from an average of 5.7% per week during the January 6, 2019, to March 15, 2020, period to 2.3% per week during the March 22, 2020, to December 19, 2021, period (Table 1, Figure 2). After April 2020, the proportion of lengthy opioid claims dropped to a new steady state, with monthly averages ranging from 1.3% to 2.9%.

### Proportion of pharmacist-prescribed claims

**Figure 3:**
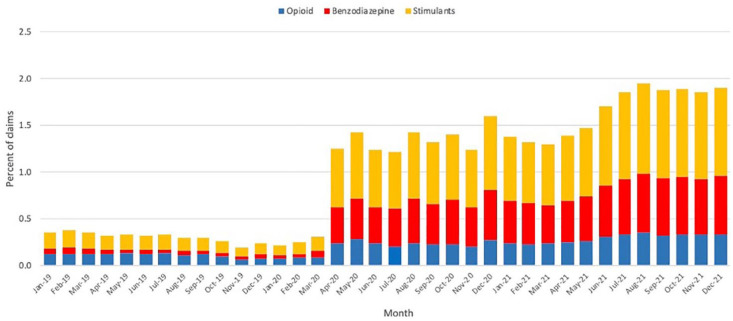
Monthly proportion of pharmacist-prescribed opioid, benzodiazepine and stimulant claims from January 2019 to December 2021

The proportion of pharmacist-prescribed opioid claims increased by 154%, from an average of 0.10% per month during the January 6, 2019, to March 15, 2020, period to 0.26% per month during the March 22, 2020, to December 19, 2021, period (Table 2, Figure 3). The proportion of stimulant claims increased by 2034%, from an average of 0.02% per month during the January 6, 2019, to March 15, 2020, period to 0.42% per month during the March 22, 2020 to December 19, 2021 period. A statistically significant increase in the proportion of pharmacist-prescribed opioid, benzodiazepine and stimulant claims was found on April 5, 2020 (*p* < 0.0001), from the ARIMA step function (Appendix 1). Between the pre- and postexemption periods, the number of total prescribed opioid and benzodiazepine claims decreased by an average of 4% (158,385 to 152,204 claims/month) and 2% (111,374 to 108,089 claims/month), respectively. The number of total prescribed stimulant claims increased by 8% (38,717 to 41,969 claims/month) (Table 2).

**Table 2:** Monthly percent change to the proportion of pharmacist-prescribed opioid, benzodiazepine and stimulant claims between pre- and postexemption periods

	Preexemption (January 6, 2019–March 15, 2020)	Postexemption (March 22, 2020–March 21, 2021)	% Change
Proportion opioid claims (%)	0.10	0.26	154
Proportion benzodiazepine claims (%)	0.043	0.48	1001
Proportion stimulant claims (%)	0.020	0.42	2034
Total opioid claims (*n*)	158,385	152,204	–4
Total benzodiazepine claims (*n*)	111,374	108,089	–3
Total stimulant claims (*n*)	38,717	41,969	8

## Discussion

This study described the impact of the CDSA exemption on pharmacist-prescribed opioid, benzodiazepine and stimulant claims in Ontario. We observed a significant increase in pharmacist-prescribed opioids, benzodiazepines and stimulants 2 weeks after the exemption was enacted. There were significant increases in the number of opioid, benzodiazepine and stimulant claims by 1.5, 9.6 and 21.5-fold, respectively. Pharmacists did not overuse the CDSA exemption: despite the large increases to pharmacist-prescribed medications, the absolute rate of pharmacist prescribing was low for these medications, as the cumulative proportion of pharmacist-prescribed claims never exceeded 2% of the total prescribed claims during the postexemption period. This may be explained as pharmacists using the exemption judiciously, pharmacist uptake of the CDSA exemption was poor (due to reluctance to use this new authority) or that it was a last resort for some. The uptake of this exemption should continue to be monitored, and the underutilization should be further explored. To optimize the use of this exemption, pharmacists may require further training or support to enhance their confidence in leveraging this expanded scope of practice. There were also lower proportions of total prescribed opioid claims that exceeded a 30-day supply seen after the CDSA exemption was enacted, indicating that longer opioid prescriptions were given out less frequently, through pharmacists or otherwise. These early results highlight the demand for pharmacist intervention during the pandemic and the potential for pharmacists to serve as the last line of defense in ensuring continuity of care, while supporting a trend towards appropriate opioid stewardship.

Prior to the enactment of the CDSA exemption, pharmacists were found to prescribe controlled substances at low rates that were likely limited to only adaptations and renewals from emergency scenarios, the Pharmaceutical Opinion Program, deprescribing initiatives or medical directives.^28-31^ After the exemption was enacted, pharmacists increasingly used the exemption to provide controlled substances as a result of shifts to virtual care and the systemic impacts of the Ontario-wide lockdown. As of 2020, 71% of all visits to a physician were conducted virtually and the number of office visits decreased by 28%.^32^ Community pharmacies were essential services that remained open throughout the lockdowns, and thus access to medication supplies remained possible for patients.^33^ Additionally, increasing numbers of patients seeking information or treatment alongside additional screening and sanitation measures for in-person appointments resulted in increased prescriber workloads, preventing timely responses to pharmacies.^34,35^ The accessibility of pharmacists in the community setting allows for timely responses in resolving prescription issues and renewals that effectively bridged the gap in care and maintained continuity of care during the pandemic.^36^ Interestingly, a periodic increase in opioid, benzodiazepine and stimulant claims was seen between December 13, 2020, and January 10, 2021, indicating that pharmacists increasingly used the exemption during this period in response to increasing demand for renewals and adaptations during holiday clinic closures (Figure 1). Due to increasing numbers of patients unable to physically visit physicians, pharmacists are becoming an essential point of contact for the vulnerable elderly and homebound population.^12^

After the exemption was enacted, a 2-week lag period (between March 22 and April 5, 2020) was observed before the rise in opioid, benzodiazepine and simulant claims that may be attributed to multiple factors. In March, many regulatory changes occurred in Ontario, tasking pharmacists with additional COVID-19 counselling, sanitation measures, managing ongoing drug shortages and adapting to new 30-day supply limitations and OAT guideline updates, in addition to clinical responsibilities.^37,38^ As the lockdown progressed, patient drug supplies dwindled and subsequent attempts to contact prescribers resulted in long patient wait times, thus requiring patients to seek out pharmacists to either contact physicians on their behalf for renewal authorizations or independently renew the prescription.^32^ Additional time was required to identify patients experiencing treatment gaps, potentially delaying pharmacist utilization of the exemption.^32^ This finding serves as an example of rapid practitioner responsiveness during systemic changes and should be considered when planning future policy changes and responses to emergency scenarios.

Despite the increase in pharmacist-prescribed claims, the cumulative proportion of these claims was always very low—less than 0.5% of the total number of opioid claims during the postexemption period, indicating that pharmacist prescribing of controlled substances was limited even when barriers to prescribing were lifted (Figure 3). General hesitation exists among health care providers and patients around pharmacist prescribing and the possibility of overuse or misuse of the new CDSA exemption.^39,40^ However, after enactment of the exemption, pharmacists were found to prescribe at low rates, and this finding, along with the persistently low proportion of opioid claims containing quantities exceeding a 30-day supply, suggests that pharmacists did not generally provide prescriptions of longer duration and stayed within the scope of the exemption. This aligns with previous work assessing pharmacist prescribing, which has found pharmacists to often be more conservative with their prescribing.^41^ This may suggest the exemption is underused, and further research may explore strategies to empower pharmacists to optimize their scope of practice.

### Limitations

This study examines a unique situation of pharmacist prescribing under an emergent policy change, with a core strength being its capture of all controlled drug prescribing at the population level in Ontario, the most populous province in Canada. Several limitations of this study arose from the data and warrant discussion. First, it is unclear if pharmacists entered their own prescriber ID or another prescriber’s ID while dispensing controlled substances, and further validation of this variable is needed. This may have underestimated pharmacist- prescribed opioid, benzodiazepine and stimulant claims. Second, sales of over-the-counter codeine were not captured, and it is unclear if patients relied on these products as bridging therapy. Additionally, this data captured the extension, adaptation and renewal capabilities of the exemption, excluding other aspects of the CDSA. We did not include opioid, benzodiazepine and stimulant indications in our analysis of the claims data or patient demographics, which may be included in future studies to better understand the specific medications and usage scenarios the exemption was applied to. Finally, patient outcomes and regional and pharmacist-specific variations were not included in this study. Further analysis is needed to identify the impact of the elevated prescribing rate on patient outcomes. Additional research is required to determine whether the patients receiving opioids, benzodiazepines and stimulants through pharmacists were less likely to discontinue therapy during the pandemic. Examining the systemic impact to pharmacy practice and apparent hesitancy of pharmacists to use the CDSA exemption during COVID-19 is essential to understand systemic barriers to patient care.

## Conclusion

This study demonstrates the impact of the CDSA exemption for pharmacists to facilitate their response to the pandemic to ensure continuity of patient care. The CDSA exemption led to increased pharmacist prescribing that remained elevated, while rates of lengthier opioid prescriptions decreased and remained low throughout 2020. These results should inform discussions of the role that pharmacists play in supporting continuity of care and medication adherence for patients and guide responses to emergent scenarios in the future. Further research is needed to investigate the relevance and impact of the CDSA exemption. ■

## Supplemental Material

sj-pdf-1-cph-10.1177_17151635221126481 – Supplemental material for Impact of the COVID-19 Controlled Drugs and Substances Act exemption on pharmacist prescribing of opioids, benzodiazepines and stimulants in Ontario: A cross-sectional time-series analysis Click here for additional data file. Supplemental material, sj-pdf-1-cph-10.1177_17151635221126481 for Impact of the COVID-19 Controlled Drugs and Substances Act exemption on pharmacist prescribing of opioids, benzodiazepines and stimulants in Ontario: A cross-sectional time-series analysis by Ann Chang, Shanzeh Chaudhry, Daniel McCormack, Tara Gomes, Anisa Shivji and Mina Tadrous in Canadian Pharmacists Journal / Revue des Pharmaciens du Canada
