# Supplementary material for: Impact of the COVID-19 Controlled Drugs and Substances Act exemption on pharmacist prescribing of opioids, benzodiazepines and stimulants in Ontario: A cross-sectional time-series analysis
Source: Can Pharm J (Ott). 2022 Oct 12;155(6):326–33. doi: 10.1177/17151635221126481 (PMC9647400; doi:10.1177/17151635221126481)
Supplement: sj-pdf-1-cph-10.1177_17151635221126481 – Supplemental material for Impact of the COVID-19 Controlled Drugs and Substances Act exemption on pharmacist prescribing of opioids, benzodiazepines and stimulants in Ontario: A cross-sectional time-series analysis [file sj-pdf-1-cph-10.1177_17151635221126481.pdf]

APPENDIX 1 ARIMA step and pulse modelling for number and proportion of pharmacist-prescribed opioid, benzodiazepine and stimulant claims (from January 2019 to Nov 22, 2020)

Parameter	Step Function (April 5, 2020)	Pulse Function (April 5, 2020)
Number opioid claims	$p < 0.0001$	$p = 0.382$
Number BZD* claims	$p < 0.0001$	$p = 0.811$
Number stimulant claims	$p < 0.0001$	$p < 0.0001$
Proportion opioid claims	$p < 0.0001$	$p = 0.282$
Proportion BZD claims	$p < 0.0001$	$p = 0.9258$
Proportion stimulant claims	$p < 0.0001$	$p = 0.0001$

* BZD, benzodiazepine.

Chang A, et al. Impact of the COVID-19 Controlled Drugs and Substances Act exemption on pharmacist prescribing of opioids, benzodiazepines and stimulants in Ontario: a cross-sectional time-series analysis. *Can Pharm J (Ott)* 2022;155. DOI: 10.1177/17151635221126481.
